# Supplementary material for: Spike Avalanches Exhibit Universal Dynamics across the Sleep-Wake Cycle
Source: PLoS One. 2010 Nov 30;5(11):e14129. doi: 10.1371/journal.pone.0014129 (PMC2994706; doi:10.1371/journal.pone.0014129)
Supplement: Figure S1 — Spike sorting and experimental design (a) The top panel shows the waveforms of two single units recorded from one electrode. The bottom panel shows that the two units can be separated as distinct clusters in a PC space. (b) The top panel shows the waveforms of multiple single units recorded from 16 channels. The bottom panel shows a rastergram of the sorted units. (c) Waveform stability was tracked throughout the experiment. Spike data (voltage-time ellipsoids, left panels) were sampled regularly from eight epochs of the total recording time (waveforms, right panels). The top left panels show good superposition of the ellipsoids, which indicates stability of a unit included in the study. The bottom left panels show discontinuity of the ellipsoids over time, indicating instability of a unit discarded from the study. (d) The FB animals were recorded across their spontaneous wakesleep cycle, comprising WK, SWS and REM. Recordings were performed before, during and after exposure to novel objects. This exposure consisted of a 20 minutes session in which four novel objects were placed inside the recording box (middle panel). Recordings made before (PRE, left panel) and after (POST, right panel) the exploration session lasted for up to 3h. Figure adapted from Ref. [23]. (0.31 MB PDF) [file pone.0014129.s001.pdf]

Spike sorting was based on waveform shape differences, peak-to-peak spike amplitudes plotted in principal component (PC) space, characteristic inter-spike-interval distributions, and a maximum 1% of spike collisions assuming a refractory period of 1 ms. Candidate spikes with signal-to-noise ratio lower than 2.5 were discarded. A waveform-tracking technique with periodic template adjustment was employed for the continuous recording of individual units over time (see Fig. S1).

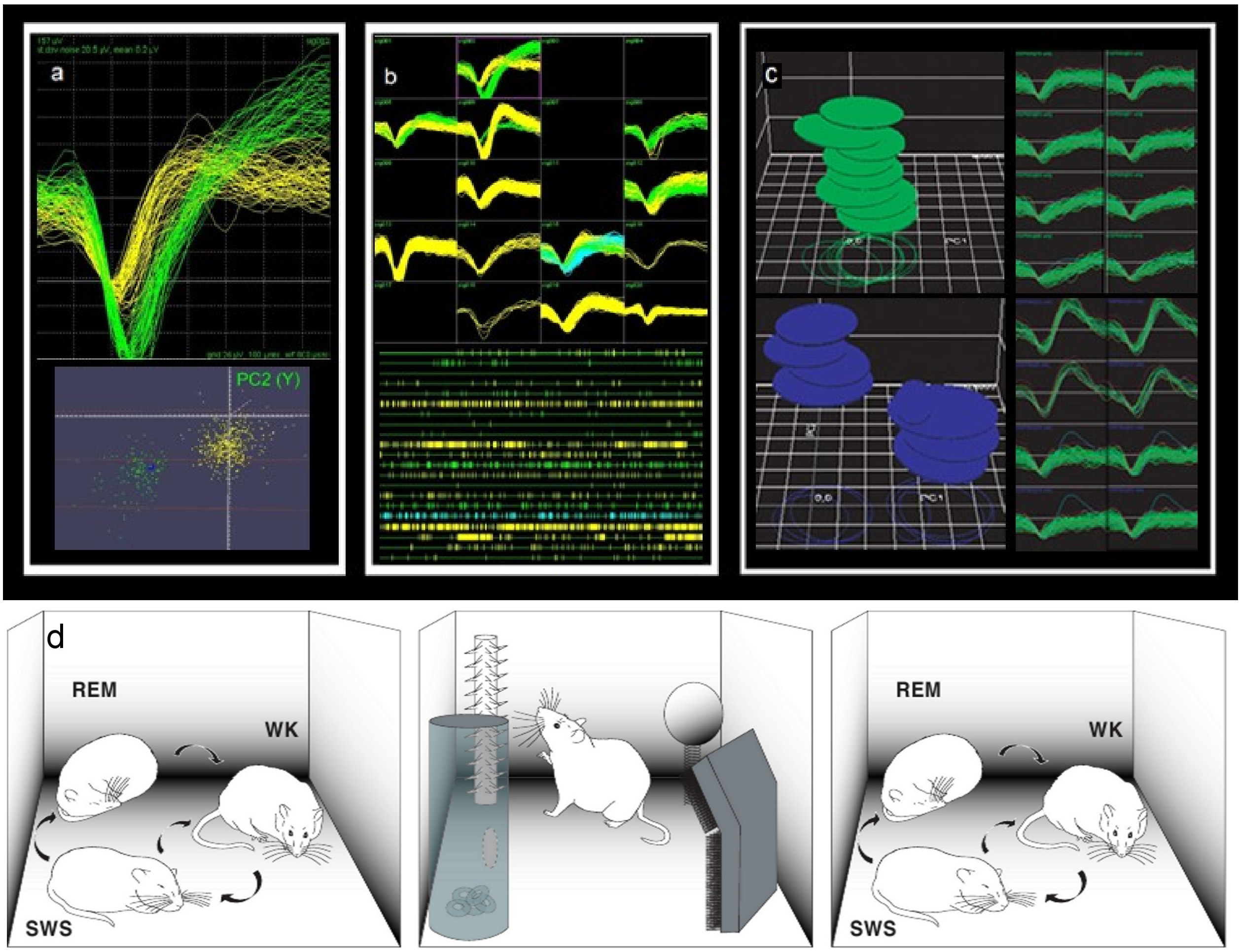

Fig. S1: Spike sorting and experimental design. (a) The top panel shows the waveforms of two single units recorded from one electrode. The bottom panel shows that the two units can be separated as distinct clusters in a PC space. (b) The top panel shows the waveforms of multiple single units recorded from 16 channels. The bottom panel shows a rastergram of the sorted units. (c) Waveform stability was tracked throughout the experiment. Spike data (voltage-time ellipsoids, left panels) were sampled regularly from eight epochs of the total recording time (waveforms, right panels). The top left panels show good superposition of the ellipsoids, which indicates stability of a unit included in the study. The bottom left panels show discontinuity of the ellipsoids over time, indicating instability of a unit discarded from the study. (d) The FB animals were recorded across their spontaneous wake-sleep cycle, comprising WK, SWS and REM. Recordings were performed before, during and after exposure to novel objects. This exposure consisted of a 20 minutes session in which four novel objects were placed inside the recording box (middle panel). Recordings made before (PRE, left panel) and after (POST, right panel) the exploration session lasted for up to 3h. Figure adapted from Ref. [23].
