# Supplementary material for: Spike Avalanches Exhibit Universal Dynamics across the Sleep-Wake Cycle
Source: PLoS One. 2010 Nov 30;5(11):e14129. doi: 10.1371/journal.pone.0014129 (PMC2994706; doi:10.1371/journal.pone.0014129)
Supplement: Figure S2 — Size distributions obtained from a decreasing number of sampled neurons in the MEA are not qualitatively different. The plots show size distributions obtained from decreasing subsamples of the total number of neurons recorded in the experiment (FB1, WK, S1, PRE). Percentages indicate the sampled fraction of the recorded neurons. Inset: Size distributions obtained from the model. (0.10 MB PDF) [file pone.0014129.s002.pdf]

## Further undersampling of FB data and model

We have deliberately discarded neurons from our analysis in order to investigate the effect of further undersampling in the system. In Fig. S2, 100% means that all neurons sampled by the MEA were considered. Decreasing this fraction to 50%, 25%, 12%, 6% and a single neuron does not change qualitatively the distributions, which are still well fit by lognormals. The model captures this relative insensitivity to undersampling fairly well (inset of Fig. S2).

Naively, one could expect a decrease in the probability of finding larger avalanches as the number of sampled neurons is reduced. However, note that less neurons lead to larger inter-event-intervals, and therefore to larger time bins, which compensate for the sparser global activity.

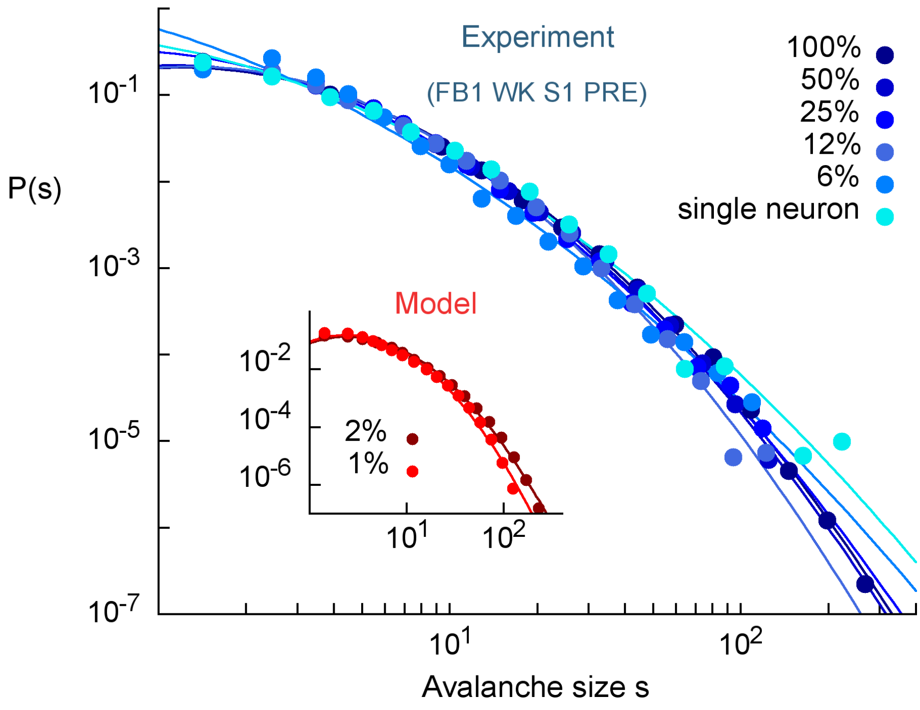


**Fig. S2: Size distributions obtained from a decreasing number of sampled neurons in the MEA are not qualitatively different.** The plots show size distributions obtained from decreasing subsamples of the total number of neurons recorded in the experiment (FB1, WK, S1, PRE). Percentages indicate the sampled fraction of the recorded neurons. Inset: Size distributions obtained from the model.
