# Supplementary material for: Spike Avalanches Exhibit Universal Dynamics across the Sleep-Wake Cycle
Source: PLoS One. 2010 Nov 30;5(11):e14129. doi: 10.1371/journal.pone.0014129 (PMC2994706; doi:10.1371/journal.pone.0014129)
Supplement: Figure S3 — Original vs surrogated FB avalanche size distributions. Comparison of the original (red) and surrogated (black) WK size distributions for different brain regions and stages of the experiment (in log-linear plots). Distributions were obtained by pooling avalanches from all FB rats. (0.13 MB PDF) [file pone.0014129.s003.pdf]

Avalanche size distributions for surrogated data

Comparison between original and surrogated avalanche size distributions shows that large avalanches occur significantly more than would be expected from uncorrelated spike trains. In Figure S3, size distributions from different stages of the experiment and brain regions are shown (in a log-linear plot), calculated from both original and surrogated data. As in Figure 2, those distributions were pooled from all FB rats. Notice the lower probability of finding large avalanches for the surrogated data sets. Likewise, distributions from surrogated AN data resulted in a reduced probability for large avalanches, when compared with the original data (Fig. S4).

Owing to these results, together with distributions obtained from AN rats, the similarity observed between size distributions obtained from different natural behaviors cannot be associated with the normalization of the bin width by the mean inter-event interval (by construction, the surrogated data had exactly the same time bin).

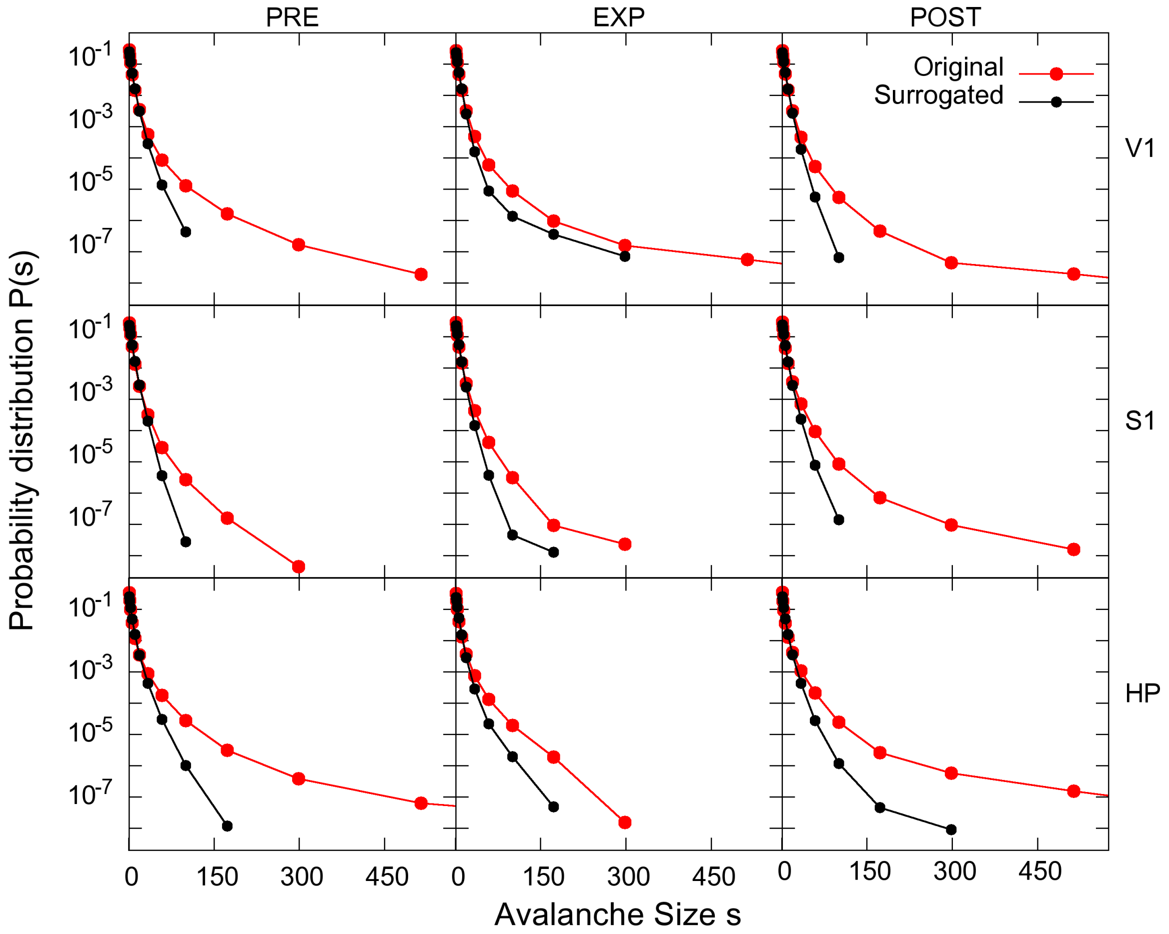

Fig. S3: Original vs surrogated FB avalanche size distributions. Comparison of the original (red) and surrogated (black) WK size distributions for different brain regions and stages of the experiment (in log-linear plots). Distributions were obtained by pooling avalanches from all FB rats.
