# Supplementary material for: Spike Avalanches Exhibit Universal Dynamics across the Sleep-Wake Cycle
Source: PLoS One. 2010 Nov 30;5(11):e14129. doi: 10.1371/journal.pone.0014129 (PMC2994706; doi:10.1371/journal.pone.0014129)
Supplement: Figure S4 — Original vs surrogated AN size distributions. Comparison of the original (red) and surrogated (black) size distributions for some of the anesthetized animals (in log-linear plots). (0.10 MB PDF) [file pone.0014129.s004.pdf]

Avalanche size distributions for surrogated data

Comparison between original and surrogated avalanche size distributions shows that large avalanches occur significantly more than would be expected from uncorrelated spike trains. In Figure S4, size distributions from different AN animals are shown (in a log-linear plot), calculated from both original and surrogated data. Notice the lower probability of finding large avalanches for the surrogated data sets. Likewise, distributions from surrogated FB data resulted in a reduced probability for large avalanches, when compared with the original data (Fig. S3).

Owing to these results, the similarity observed between size distributions obtained from different natural behaviors cannot be associated with the normalization of the bin width by the mean inter-event interval (by construction, the surrogated data had exactly the same time bin).

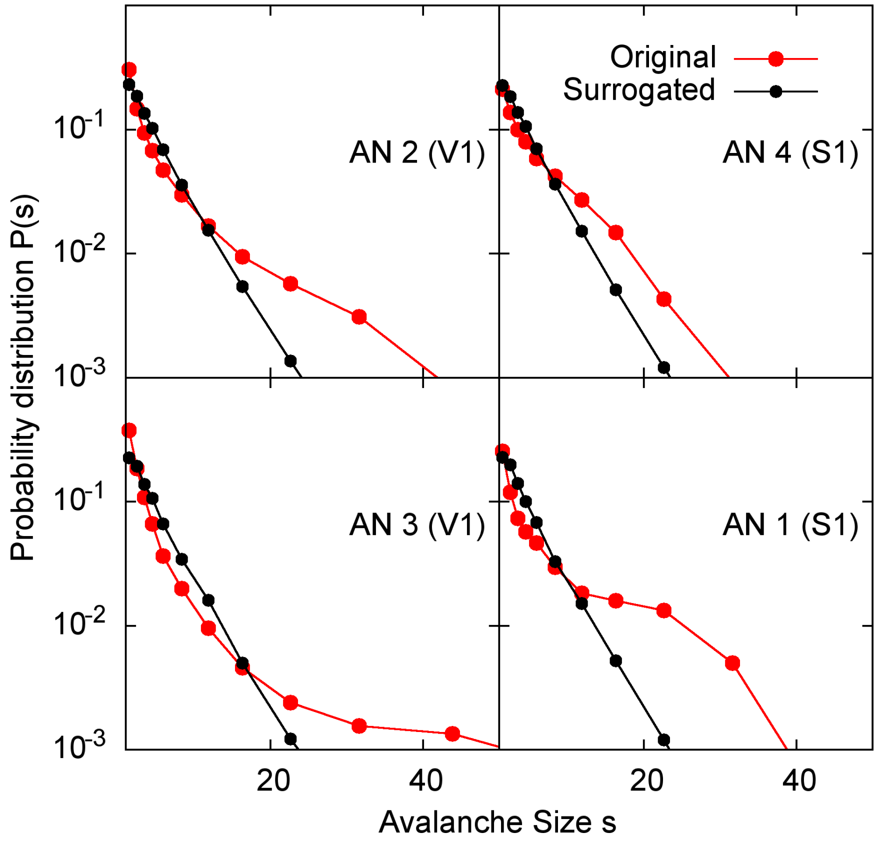

Fig. S4: Original vs surrogated AN size distributions. Comparison of the original (red) and surrogated (black) size distributions for some of the anesthetized animals (in log-linear plots).
