# Supplementary material for: Spike Avalanches Exhibit Universal Dynamics across the Sleep-Wake Cycle
Source: PLoS One. 2010 Nov 30;5(11):e14129. doi: 10.1371/journal.pone.0014129 (PMC2994706; doi:10.1371/journal.pone.0014129)
Supplement: Figure S5 — Comparison of FB cumulative avalanche size distributions for different states and stages of the experiment. Cumulative distributions are shown together with the p values calculated from the KS tests. Note that the distributions are very similar in all cases, but only the ones in the left column pass the KS test. (0.11 MB PDF) [file pone.0014129.s005.pdf]

Size distributions are similar regardless of behavioral state or stage of the experiment.

See Supporting Information Text S1 for detailed statistical analysis.

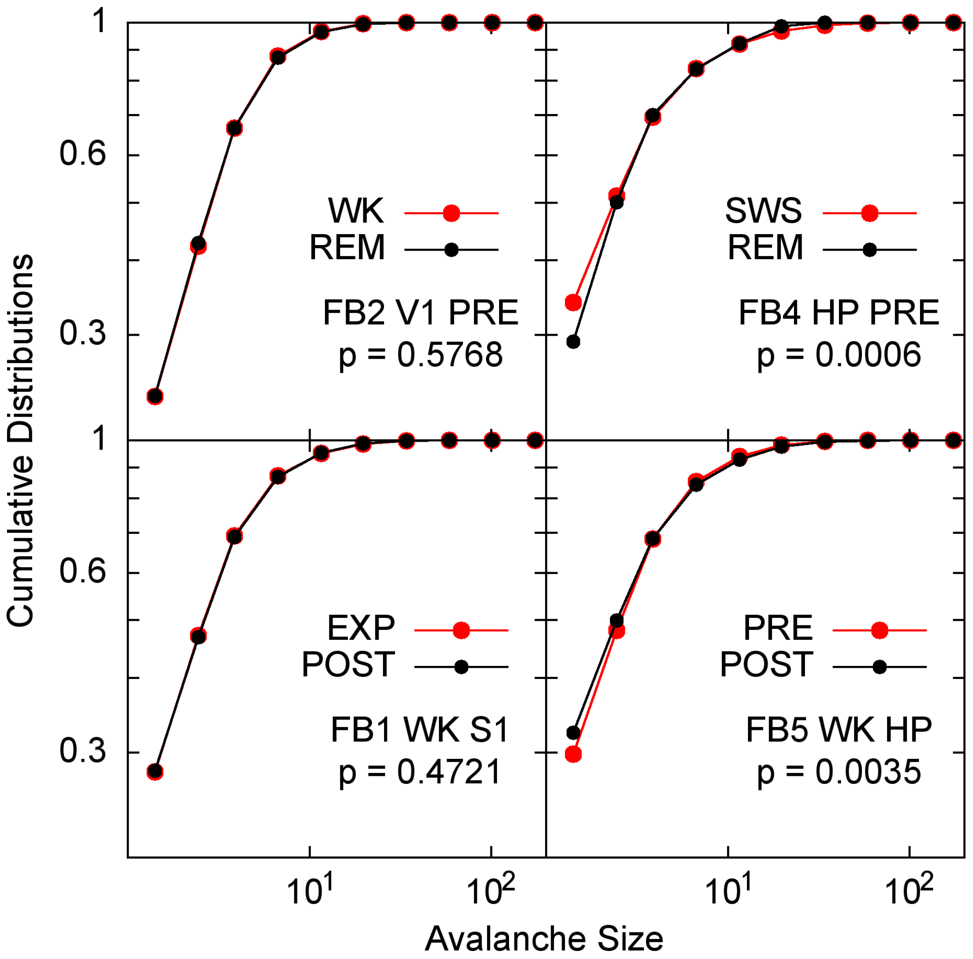

Fig. S5: Comparison of FB cumulative avalanche size distributions for different states and stages of the experiment. Cumulative distributions are shown together with the *p*-values calculated from the KS tests. Note that the distributions are very similar in all cases, but only the ones in the left column pass the KS test.
