# Supplementary material for: Spike Avalanches Exhibit Universal Dynamics across the Sleep-Wake Cycle
Source: PLoS One. 2010 Nov 30;5(11):e14129. doi: 10.1371/journal.pone.0014129 (PMC2994706; doi:10.1371/journal.pone.0014129)
Supplement: Figure S6 — Size distribution from AN data and the truncated power-law. (a) Avalanche size distribution for one AN rat and the truncated power-law fit (see text). (b) QQ-plot for the same AN data and fit. The solid line represents the linear fit which resulted in the slope a = 1. (0.11 MB PDF) [file pone.0014129.s006.pdf]

Size distributions from anesthetized animals are similar to truncated power-laws.

See Supporting Information Text S1 for detailed statistical analysis.

In Fig. S6a, we show the fit of the truncated power law. The Q-Q plot in Fig. S6b compares the data versus the fitted function by plotting their quantiles against each other. A perfect fit should yield a straight Q-Q plot with slope a=1, so we calculated the linear correlation coefficient R and the best-fitted slope a to assess the goodness-of-fit. We obtained R=0.99 and a=1.00, which indicates that the truncated power law provided indeed a good fit to the data.

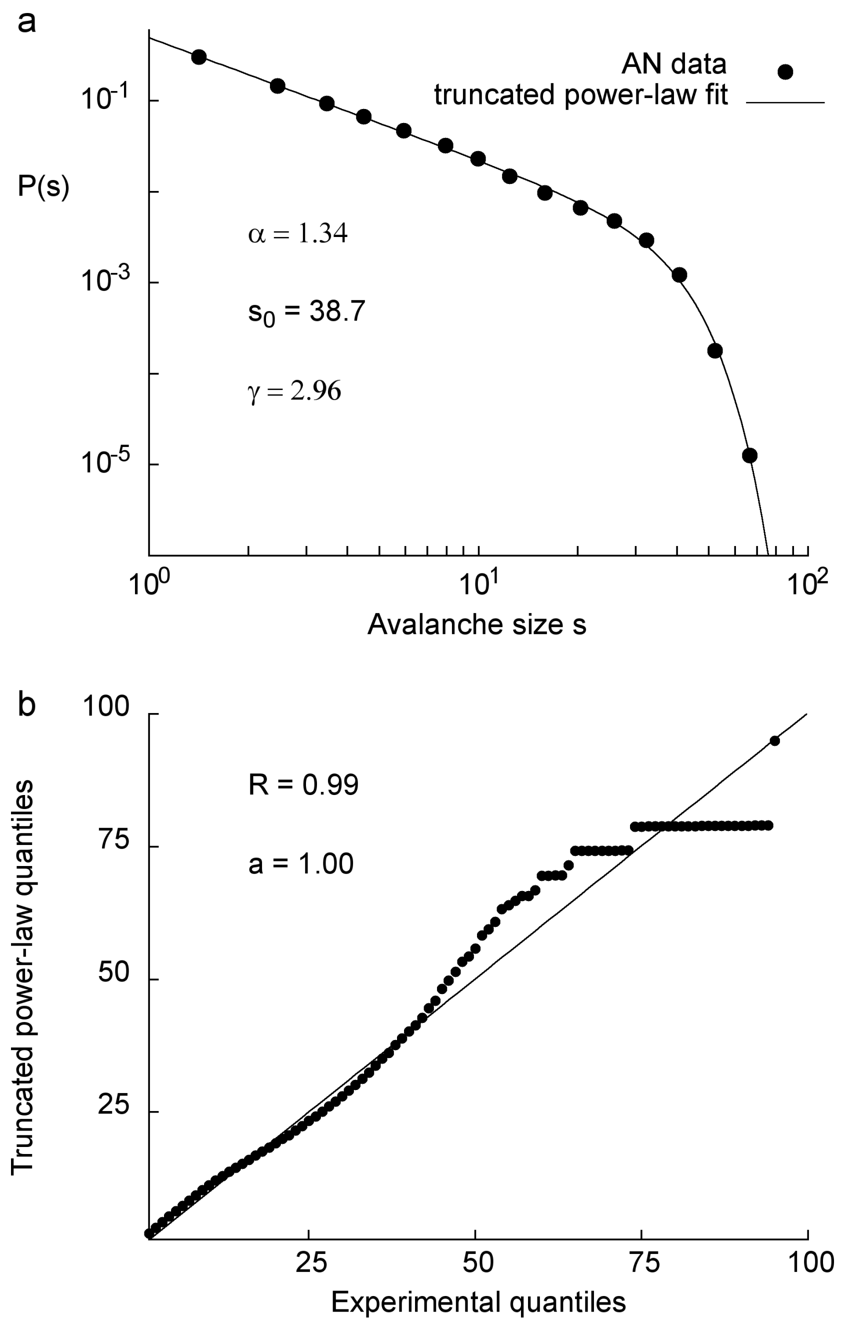

S6: Size distribution from AN data and the truncated power-law. (a) Avalanche size distribution for one AN rat and the truncated power-law fit (see text). (b) QQ-plot for the same AN data and fit. The solid line represents the linear fit which resulted in the slope a=1.
