# Supplementary material for: Spike Avalanches Exhibit Universal Dynamics across the Sleep-Wake Cycle
Source: PLoS One. 2010 Nov 30;5(11):e14129. doi: 10.1371/journal.pone.0014129 (PMC2994706; doi:10.1371/journal.pone.0014129)
Supplement: Table S1 — Time bin Δt, in milliseconds, calculated in each case. Since Δt is the mean interevent interval for a given condition, smaller sets of neurons typically lead to larger time bins (see also Table S2). (0.06 MB PDF) [file pone.0014129.s007.pdf]

PRE									
Rat	WK			SWS			REM		
	HP	S1	V1	HP	S1	V1	HP	S1	V1
FB1	6.98	-	-	10.14	-	-	8.63	-	-
FB2	47.62	4.82	3.25	21.64	6.69	4.52	19.23	5.82	3.49
FB3	43.29	11.31	4.33	44.84	18.35	7.26	49.5	15.58	6.16
FB4	6.97	8.14	5.59	6.18	10.71	7.58	6.29	7.75	5.39
FB5	3.74	1.95	9.35	4.52	3.59	19.08	3.89	2.58	13.57
FB6	3.63	2.92	4.73	10.76	3.33	8.76	-	-	-
FB7	2.08	1.31	2.04	4.27	3.12	4.71	2.98	2.71	3.18
EXP									
Rat	WK			SWS			REM		
	HP	S1	V1	HP	S1	V1	HP	S1	V1
FB1	4.13	1.7	-	-	-	-	3.88	1.46	-
FB2	24.51	2.85	2.34	-	-	-	18.05	2.91	2.1
FB3	33.56	6.39	3.07	-	-	-	-	-	-
FB4	6.02	3.58	3.01	-	-	-	-	-	-
FB5	2.42	1.58	12.69	4.16	2.81	58.14	2.99	2.06	18.28
FB6	2.51	2.78	4.31	8.9	3.29	7.67	-	-	-
FB7	1.87	1.34	2.08	2.19	1.67	3.43	-	-	-
POST									
Rat	WK			SWS			REM		
	HP	S1	V1	HP	S1	V1	HP	S1	V1
FB1	6.6	2.56	-	8.63	3.51	-	5.44	2.34	-
FB2	36.36	4.2	2.82	21.55	5.44	3.86	21.5	4.52	3.2
FB3	47.62	9.54	4.08	46.95	14.1	7.09	50	12	5.94
FB4	5.44	4.78	3	-	-	-	5.4	5.7	3.72
FB5	3.34	1.96	20.83	4.26	2.52	37.74	3.92	2.7	29.5
FB6	2.29	2.4	3.33	9.98	3.08	7.84	-	-	-
FB7	2.12	1.6	2.27	3.66	2.96	4.57	-	-	-
Anesthesia									
Rat	S1	V1	Rat	S1	V1	Rat	S1	V1	
AN1	-	12.57	AN2	-	27.03	AN3	-	20.79	
AN4	17.24	13.05	AN5	23.53	19.12	AN6	59.17	34.84	
AN7	31.65	126.58	-	-	-	-	-	-	

Table S1: Time bin Δt, in milliseconds, calculated in each case. Since Δt is the mean inter-event interval for a given condition, smaller sets of neurons typically lead to larger time bins (see also Table S2).
