# Supplementary material for: Spike Avalanches Exhibit Universal Dynamics across the Sleep-Wake Cycle
Source: PLoS One. 2010 Nov 30;5(11):e14129. doi: 10.1371/journal.pone.0014129 (PMC2994706; doi:10.1371/journal.pone.0014129)
Supplement: Table S2 — Number of neurons sampled by the MEA per brain region for FB and AN rats. (0.04 MB PDF) [file pone.0014129.s008.pdf]

Freely-behaving					Anesthetized			
Rat	HP	S1	V1	Total	Rat	S1	V1	Total
FB1	14	42	0	56	AN1	0	55	55
FB2	4	23	38	65	AN2	0	33	33
FB3	4	13	28	45	AN3	0	45	45
FB4	13	16	22	51	AN4	59	29	88
FB5	22	28	7	57	AN5	31	44	75
FB6	34	25	23	82	AN6	15	17	32
FB7	45	39	42	126	AN7	27	11	38

Table S2: Number of neurons sampled by the MEA per brain region for FB and AN rats.
