# Supplementary material for: Spike Avalanches Exhibit Universal Dynamics across the Sleep-Wake Cycle
Source: PLoS One. 2010 Nov 30;5(11):e14129. doi: 10.1371/journal.pone.0014129 (PMC2994706; doi:10.1371/journal.pone.0014129)
Supplement: Text S1 — Statistical analysis of avalanche size distributions. (0.08 MB PDF) [file pone.0014129.s009.pdf]

Statistical analysis of avalanche size distributions

One should not expect a perfect agreement between measured distributions and fitted functions, since there is a very large variation in the sampling conditions of spike avalanches owing, among other reasons, to the different durations of behavioral states, as well as variations in the amount of neurons recorded per region and per animal. Given this scenario, what is most relevant is to test whether or not the distributions are heavy-tailed. To assess the statistical significance of this and other claims (see below), we performed the Kolmogorov-Smirnov (KS) test. In all cases considered, the null hypothesis was rejected at level $p = 0.05$.

1) Size distributions are not well fit by exponentials.

In a previous study [20], spike avalanches measured from the parietal cortex of cats had their size distributions compared to an exponential function. This contrasts with our claim that the size distributions are heavy-tailed. A KS comparison of the experimental size distributions with the best-fitted exponential distributions leads to a rejection for *all* distributions in any scenario, both in the freely-behaving and in the anesthetized group. This indicates that the size distributions of the spike avalanches recorded in our study are not exponential.

2) Size distributions are similar regardless of behavioral state or stage of the experiment.

For a comparison of distributions across the different stages of the experiment (PRE, EXP and POST for the same animal and same behavioral state), the KS test reveals that 36% of the distribution pairs presented a *p*-value greater than 0.05, with a null hypothesis that the distributions are identical. When comparing distributions across the different behavioral states (WK, SWS and REM for the same animal and stage of the experiment), 22% of the distribution pairs passed the KS test by the same criterion. Figure S5 shows the cumulative probabilities compared for some cases. Note that even in the cases in which the KS test results in the distributions not being the same, they are still very similar.

3) Size distributions from freely-behaving animals are compatible with lognormals.

We performed a goodness-of-fit test to the adjusted lognormals (pooled size distributions from freely-behaving animals, see Fig. 2). The KS test resulted in 23 out of 27 distributions compatible with the fitted lognormals, and none compatible with a power law or an exponential. The surrogated data size distributions also showed a good agreement with lognormals: 21 out of 27 were compatible with the fitted lognormals. The difference between the original and surrogated data sets is that the probability for large avalanches is always smaller for the surrogated size distributions.

4) Size distributions from anesthetized animals are similar to truncated power-laws.

Following the same procedure adopted for the freely-behaving animals, we tested whether

power-laws, lognormals or exponentials represent a good fit for the size distributions of the anesthetized animals. The KS test yielded $p = 0$ for all of these distributions, whereas size distributions for surrogated AN data (Fig. S4) were compatible with the exponential fits (p > 0.05 in all cases). Since KS was not able to determine which of the tested distributions best fits the data, we employed two alternative approaches.

First, we compared the different fits using the log likelihood ratio (LLR), which gives an estimation of how well the data are described by the fitted distribution. The sign of the LLR indicates which distribution is the best fit. We chose three models to fit the data: an exponential, a lognormal and a truncated power law distribution, $P(s) \sim s^{-\alpha} \exp[-(s/s_0)^{\gamma}]$ (the exponential term fits the cutoff region, observed for sizes larger than the number of electrodes [1]). This distribution essentially behaves as a power law for $s < s_0$ and decays faster than exponentially for $s > s_0$ and $\gamma > 1$.

Calculating the LLR between the truncated power law and the exponential we obtained -9837.4, while the LLR between the truncated power-law and the lognormal yielded -13679.8. These negative numbers mean that the truncated power law fit is better than both the exponential and lognormal fits. The significance of the results can be evaluated by a *p*-value between 0 and 1. The closer to zero this value is, the less likely it is that the sign of the LLR is a consequence of random fluctuations. Both LLRs resulted in a *p*-value = 0.

Next, we attempted to evaluate the goodness-of-fit for the truncated power law. In Fig. S6a, we show the fit of the truncated power law. The Q-Q plot in Fig. S6b compares the data versus the fitted function by plotting their quantiles against each other. A perfect fit should yield a straight Q-Q plot with slope $a = 1$, so we calculated the linear correlation coefficient R and the best-fitted slope a to assess the goodness-of-fit. We obtained R=0.99 and a=1.00, which indicates that the truncated power law provided indeed a good fit to the data.
